# Frailty and Cognitive Impairment in Predicting Mortality Among Oldest-Old People

**DOI:** 10.3389/fnagi.2018.00295

**Published:** 2018-10-18

**Authors:** Qiukui Hao, Birong Dong, Ming Yang, Biao Dong, Yuquan Wei

**Affiliations:** ^1^The Center of Gerontology and Geriatrics/National Clinical Research Center for Geriatrics, West China Hospital, Sichuan University, Chengdu, China; ^2^Key Laboratory of Biotherapy and Cancer Center/Collaborative Innovation Center for Biotherapy, West China Hospital, Sichuan University, Chengdu, China

**Keywords:** frailty and cognitive impairment, frailty index, mortality, MMSE, oldest-old, cognitive frailty

## Abstract

**Backgrounds:** Frailty and cognitive impairment are critical geriatric syndromes. In previous studies, both conditions have been identified in old-age adults as increased risk factors for mortality. However, the combined effect of these two syndromes in predicting mortality among people with advanced age is not well understood. Thus, we used Chinese community cohort to determine the impact of the combined syndromes on the oldest-old people.

**Methods:** Our present study is part of an ongoing project on Longevity and Aging in Dujiangyan, which is a community study on a 90+ year cohort in Sichuan Province in China. Participants were elderly people who completed baseline health assessment in 2005 followed by a collection of mortality data in 2009. Frailty and cognitive function were assessed with 34-item Rockwood Frailty Index and the Mini-Mental Status Examination, respectively, and the combined effect(s) of these two parameters on death was examined using the Cox proportional hazard regression model.

**Results:** This study consisted of a total of 705 participants (age = 93.6 ± 3.3 years; 67.4% females), of which 53.8% died during a four-year follow-up period. The prevalence of frailty, cognitive impairment, and the overlap of these two syndromes was 63.7, 74.2, and 50.3%, respectively. Our data showed that the subjects with combined frailty and cognitive impairment were associated with increased risk of death (age, gender, education level, and other potential confounders adjusted); the hazard ratio was 2.13 (95% confidence interval 1.39, 3.24), compared with the control group. However, neither frailty alone nor cognitive impairment alone increased the risk of death in these individuals.

**Conclusion:** The combined frailty and cognitive impairment, other than the independently measured syndromes (frailty or cognitive impairment alone), was a significant risk factor for death among the oldest-old Chinese people.

## Introduction

Frailty is a common geriatric syndrome, presenting a clinical state of decreased physiological reserve and increased vulnerability to death and/or developing an increased dependency to even a small stressor (Morley et al., 2013). The prevalence of frailty is about 7.0% among community-dwelling people aged 65 years or more; it varies with different operational definitions and increasing age (Fried et al., 2001; Collard et al., 2012). Frailty is an emerging public problem with the advent of aging society worldwide, for it can increase the risk of adverse clinical outcomes, such as disability, delirium, falls, and death (Clegg et al., 2013; Cesari et al., 2016). Frailty is a transitional and reversible state, and therefore, it has provided us with an opportunity to carry out research which would provide insight into the occurrences and consequences “of adverse outcomes” among the elderly and to plan strategies to reduce the incidence of any non-reversible adverse outcomes (Michel et al., 2015). Currently, the specific pathophysiology of frailty is poorly understood, and the frailty state has generally been regarded as a disorder of several physiological systems, including the brain, skeletal muscle, endocrine system, and the immune system (Clegg et al., 2013).

Brain aging or frail brain plays an essential role in physical frailty (Malmstrom and Morley, 2013). More and more studies have shown frailty to be closely related to cognitive impairment in a prospective cohort study (Boyle et al., 2010; Sugimoto et al., 2018). In order to encourage combined research in frailty and cognitive impairment, the International Academy on Nutrition and Aging (IANA) and the International Association of Gerontology and Geriatrics (IAGG) organized an International Consensus Group (ICG) to propose the operational definition of cognitive frailty (Kelaiditi et al., 2013). After the consensus was published, researchers put more attention on these two critical geriatric syndromes. Although the prevalence of cognitive frailty in the community setting is low (1.0–1.8%), it has been associated with a high risk of disability, poor quality of life, and death (Sugimoto et al., 2018). Furthermore, researchers have also found a 50-item frailty index (FI) to be significantly associated with temporal and frontal cortical atrophy, detected by computerized axial tomography, which indicates that frailty and cognitive decline might share common pathophysiological mechanisms (Fougere et al., 2017; Gallucci et al., 2018). All of these findings show that frailty and cognitive impairment are closely related to each other. However, Shimada et al. (2013) found that only 2.7% participants displayed overlapping frailty and cognitive impairment, with the majority of the subjects (97.3%) devoid of the combined syndromes.

The use of frailty and cognitive impairment parameters in predicting mortality has previously been investigated (Jacobs et al., 2011; Matusik et al., 2012; Forti et al., 2014; Jha et al., 2016; Feng et al., 2017b; Lee et al., 2018). The results, however, revealed several discrepancies among various reports; some found combined physical frailty and cognitive function assessment to enhance the likelihood of the prediction of individual’s risk of death than either measurement alone (Matusik et al., 2012; Jha et al., 2016; Feng et al., 2017b; Lee et al., 2018), while some researchers found no statistically significant enhancement of the combined effect (Jacobs et al., 2011; Forti et al., 2014). Furthermore, most participants in these studies were Caucasians and aged from 60 to 90 years. The characteristic of cognition or frailty among the oldest-old had been shown to be different with other age groups (Luo et al., 2013; Hao et al., 2016). Thus, the role of cognitive impairment, frailty or a combination of both in predicting adverse outcomes need to be further classified, primarily, among the oldest-old (aged 90 or more) and also other races. Based on the above-mentioned findings, we hypothesized that the combined effect(s) of cognitive impairment and frailty would be more capable of predicting mortality in very old Chinese people than the independent syndromes.

To date, no studies have focused on only cognitive impairment or frailty or the two syndromes combined in predicting mortality in advance late-life, and thus the combined effects remain unclear in the oldest-old population (90+ years or older). In 2005, we included 870 old-aged people (aged 90 years or older) in Dujiangyan (town level), Chengdu, and Sichuan in China, for the PLAD project explained in detail in the Methods section below. Four years later (in 2009) we collected the information on the death of the participants (4-year all-cause mortality and the time of death). This study provided us with the opportunity to explore the effects of frailty and cognitive impairment or a combination of both in predicting mortality in this elderly population.

## Materials and Methods

### Study Population

The data from the Project of Longevity and Aging in Dujiangyan (PLAD) is a cross-sectional study conducted in Dujiangyan in 2005. Dujiangyan is a town of Chengdu located in southwestern China. PLAD was conducted to explore the relationship between age-related diseases, longevity, lifestyle, and other factors. The details regarding the PLAD research have been reported previously (Wu et al., 2007; Wang et al., 2010; Flaherty et al., 2011). Briefly, PLAD included 870 elderly people aged 90 years or older, based on the 2005 census in Dujiangyan region (total of 1115 community members, aged 90 years or older). Face-to-face interviews with trained volunteers were used to collect baseline data, using several validated scales of general questionnaires. Medical staff performed anthropometric measurements, physical examination, and collection of fasting blood samples for various analyses [22–24]. All the participants or their legal proxies were informed about the details of the study and gave formal written consent before the study was initiated. The Ethics Committee of Sichuan University approved the study protocol (Chengdu, Sichuan, China). The exclusion criteria for our current study were as follows: participants with missed data on mortality (*n* = 53), MMSE (*n* = 100), or >20% of the FI variables (*n* = 10), and participants with previous denoised dementia (*n* = 2), which resulted in a study population of 705 (males: 230 cases or 32.6%; females: 475 cases or 67.4%).

### Construction of the Frailty Index

In this study, FI was constructed using 34 items available in the PLAD dataset, according to a standard procedure, which was similar to previous study reports (Searle et al., 2008; Hao et al., 2016). All selected variables meet the following criteria: associated with health status; increased with age (generally); not saturate too early; cover a range of important systems (Searle et al., 2008). The 34 variables in the construction of FI were Instrumental Activities Daily Living (IADL) and Activities Daily Living (ADL) disability items (*n* = 14), disease (*n* = 9), psychological problems (*n* = 1), symptoms (*n* = 5), and abnormality in the physical examination (*n* = 6). Items used to assess the cognitive function such as all items in MMSE were excluded. A binary variable was coded as present = 1 or absent = 0. For variables with 3–4 scale levels, the intermediate response was coded between 0 and 1. For each old-age person, the FI was calculated as the sum of all deficits present divided by the total number of whole considered variables (here it is 34), which made the FI a continuous variable, theoretically ranging between 0 and 1. We set FI = 0.21 as a cut-off point for diagnosis of frailty, in accordance with Hoover et al. (2013) study.

### Evaluation of Cognitive Function

In this study, the 30-item Mini-Mental State Examination (MMSE) scale was used to evaluate cognitive function, as it is a reliable and widely used method of assessment of the condition, and it includes the measurements of the following parameters: attention and calculation, orientation, recall, language, and ability to follow simple commands (Tuijl et al., 2012). Visual and auditory abilities were basic requirements for most items of MMSE (Holtsberg et al., 1995). Our study excluded 100 participants (28 men and 72 women) who were unable to complete the MMSE test due to hearing or visual problems, in order to be able to address the influence of hearing and visual impairment on cognitive function. In Asian people, the cut-off point of MMSE is highly variated, ranging from 17 to 29 (Rosli et al., 2016). The educational level of most subjects in this study was low (illiterate or primary school; 97.4%), and cognitive impairment was defined as an MMSE score of 0–18. An MMSE score of 19–30 was defined as “without cognitive impairment,” according to previous reports, and this cutoff point has been shown to be 80 to 100% specific and 80 to 90% sensitive for diagnosis of cognitive impairment (Katzman et al., 1988; Tombaugh and McIntyre, 1992; Zhu et al., 2006; Cui et al., 2011; Matusik et al., 2012). Furthermore, we performed several methods to promote the assessment quality and methodological reliability, which includes the following: (1) MMSE assessors were trained by experienced geriatricians in comprehensive geriatric assessment, provided research manually, and video for all researchers; (2) observed MMSE administrators performing the MMSE on standardized patients; (3) quality control researchers received and responded to feedback or questions, while conducting the MMSE on the participants.

### Mortality Data and Other Co-variables

The mortality data, the status of survival (died or survived), and the time of death, were collected for all participants from local government records, relatives, or neighbors in 2009. There were about 48 (5.5%) participants lost to follow-up. Age, gender, educational levels (illiteracy, primary school, or secondary school and advanced), weight, height, waist circumference, systolic blood pressure, diastolic blood pressure, smoking, alcohol drinking, exercise, and comorbidity were collected as co-variables. Comorbidity was defined as two or more chronic illnesses occurring in the same participant. All chronic diseases were diagnosed by certified physicians in the local hospital.

### Statistical Analysis

In this study, baseline characteristics of the participants were shown according to the status of frailty and cognitive impairment of the data types. Continuous variables were presented as means and standard deviations. Categorical variables were presented as numerals and percentages. The differences between groups were tested by Analysis of Variance (ANOVA) or unpaired Student’s *t*-test for continuous variables or Chi-square test for categorical variables. Cox proportional hazard regression models were employed to estimate the hazard ratio (HR) and its 95% confidence interval (CI) of the status of frailty and cognitive impairment as a function of increased mortality. Age, gender, and educational levels were regarded as general covariates in adjusted Cox regression model 1. Lifestyle factors (smoking, alcohol consumption, and exercise) and chronic diseases were added in Cox regression model 2. Several other co-variables (*P* < 0.1, when compared among different groups for baseline variables), regarded as potential confounders, were adjusted further in model 3. Statistical Product and Service Solutions (SPSS) software package for Windows, version 17.0 (SPSS Inc., Chicago, IL, United States), was used in all statistical analyses. Two-tailed *P*-values of <0.05 were set as statistically significant.

## Results

### Baseline Characteristics, Frailty, and Cognitive Impairment

Overall, we included 705 participants in this study. The percentage of females was 67.4%, and the mean age of the subjects was 93.6 ± 3.3 years, ranging from 90 to 108. The maximum, mean, and median FI scores of the participants were 0.62, 0.26, and 0.25, respectively. The standard deviation of FI is 0.10. The 99th percentile obtained for the FI was 0.53. The maximum, mean, and median MMSE scores of the participants were 28, 14.82, and 15, respectively. The standard deviation of MMSE was 5.68.

Women had significantly higher FI scores and lower MMSE scores than men (0.26 ± 0.11 vs. 0.24 ± 0.10; *t* = -2.53, *P* = 0.012; 13.70 ± 5.28 vs. 17.14 ± 5.79; *t* = 7.86, *P* < 0.001) and more females presented in the frailty and cognitive group than males (66.1 vs. 58.7%, *X*^2^ = 3.68, *P* = 0.055; 83.6 vs. 54.8%, *X*^2^ = 67.01, *P* < 0.001). The overall prevalence of frailty and cognitive impairment among the whole population were 63.7% (95% confidence interval (CI) = 60.1–67.2%) and 74.2% (95% CI = 70.8–77.3%), respectively.

The combined prevalence of frailty and cognitive impairment, frailty alone, cognitive impairment alone, and no frailty nor cognitive impairment (control group) were 50.1% (95% CI = 46.4–53.8%), 13.6% (95% CI = 11.3–16.4%), 24.1% (95% CI = 21.1–27.4%), and 12.2% (95% CI = 10.0–14.8%), respectively. Subjects with combined frailty and cognitive impairment were older with significantly higher percentage of female, illiteracy, comorbidity, and death, but significantly lower weight, height, and systolic blood pressure (SBP), compared with the control group. **Table 1** shows the characteristics of the study participants, according to their frailty and cognitive impairment status.

**Table 1:** Characteristics of the study population according to frailty and cognitive impairment status.

	Status of frailty and cognitive function				
	Frailty and cognitive	Frailty only	Cognitive impairment only	No frailty and no cognitive	*P-*value
	impairment jointly	(*n* = 96)	(*n* = 170)	impairment (control group)	
	(*n* = 353)			(*n* = 86)	
Age (years)	94.2 @ 3.5	93.1 @ 3.4	93.3 @ 3.1	92.5 @ 2.6	<0.001**
Female (%)	75.1	51.0	77.6	33.7	<0.001**
BMI (kg/m^2^)	18.9 @ 3.5	19.4 @ 4.2	19.5 @ 2.9	20.2 @ 3.3	0.009**
Weight (kg)	39.7 @ 7.9	41.9 @ 9.4	41.3 @ 7.3	46.7 @ 9.3	<0.001**
Height (cm)	145.4 @ 10.5	147.9 @ 10.6	145.4 @ 8.6	152.5 @ 7.7	<0.001**
WC (cm)	76.7 @ 9.2	76.5 @ 11.1	77.1 @ 8.5	79.3 @ 9.8	0.131
SBP (mmHg)	137.3 @ 22.8	144.0 @ 22.6	144.4 @ 23.4	140.8 @ 23.0	0.003**
DBP (mmHg)	72.6 @ 12.0	73.4 @ 12.4	72.8 @ 12.1	73.0 @ 12.1	0.936
MMSE	11.9 @ 4.5	21.4 @ 2.0	13.5 @ 3.9	22.2 @ 2.4	<0.001**
Frailty index	0.32 @ 0.08	0.29 @ 0.06	0.15 @ 0.04	0.16 @ 0.04	<0.001**
**Education level (%)**					
Illiteracy	84.4	49.5	80.6	31.8	
Primary school	13.9	47.4	17.1	62.4	
Secondary school or advanced	1.7	3.2	2.4	5.9	<0.001**
Smoking (%)	40.3	50.0	46.2	44.2	0.311
Alcohol drinking (%)	21.7	22.9	29.6	41.9	0.001**
Having exercise habit (%)	36.0	36.8	42.5	51.2	0.056
Comorbidity (%)	77.3	76.0	17.6	29.1	<0.001**
**Status of survival (%)**					
Alive	39.7	52.1	50.0	59.3	
Death	60.3	47.9	50.0	40.7	0.002**


*Data are the mean ± SD unless otherwise indicated. Comorbidity was defined as the presence of two or more chronic diseases (hypertension, cardiovascular disease, cerebrovascular disease, diabetes, respiratory disease, digestive disease, chronic renal disease, and osteoarthritis). Abbreviations: BMI, body mass index; WC, waist circumference; SBP, systolic blood pressure; DBP, diastolic blood pressure; MMSE, mini-mental status examination. ^∗∗^*P* < 0.01.*

### Baseline Characteristics and All-Cause Mortality

The 4-year death rate was 53.8% in these old-aged individuals. Those who died were slightly older than the survival group, but there was no statistical significance (93.8 ± 3.3 vs. 93.4 ± 3.4, *t* = -1.86, *P* = 0.063). Mortality was significantly enhanced in participants with higher FI but lower MMSE scores than the survival group (0.27 ± 0.11 vs. 0.24 ± 0.10, *t* = -4.32, *P* < 0.001; 13.86 ± 5.83 vs. 15.94 ± 5.31, *t* = 4.93, *P* < 0.001). The proportion of frailty and cognitive impairment was also higher in the death group than in the survival group (68.3 vs. 58.3%, *X*^2^ = 7.66, *P* = 0.006; 78.6 vs. 69.0%, *X*^2^ = 8.45, *P* = 0.004). Lifestyle habit, regular exercise, was also less common in the death group than in the survival group (33.0 vs. 47.2%, *X*^2^ = 14.63, *P* < 0.001). There was no statistically significant difference between the death and the survival groups for comorbidity (57.3 vs. 56.4%, *X*^2^ = 0.047, *P* = 0.828) and other co-variables (see **Table 2** for more details).

**Table 2:** Characteristics of the study population according to survival status.

	Status of survival		
	Alive (*n* = 326)	Death (*n* = 379)	*P-*value
Age (years)	93.4 @ 3.4	93.8 @ 3.3	0.063
Female (%)	68.4	66.5	0.589
BMI (kg/m^2^)	19.4 @ 3.3	19.2 @ 3.6	0.329
Weight (kg)	41.4 @ 8.2	41.2 @ 8.7	0.692
Height (cm)	146.6 @ 10.1	146.5 @ 10.0	0.891
WC (cm)	76.9 @ 9.7	77.2 @ 9.2	0.701
SBP (mmHg)	140.3 @ 22.5	140.4 @ 23.6	0.929
DBP (mmHg)	72.2 @ 11.4	73.3 @ 12.6	0.249
MMSE	15.9 @ 5.3	13.9 @ 5.8	<0.001**
Frailty index	0.24 @ 0.10	0.27 @ 0.11	<0.001**
**Education level (%)**			
Illiteracy	72.0	72.8	
Primary school	25.5	24.6	
Secondary school or advanced	2.5	2.6	0.952
Smoking (%)	45.7	41.6	0.279
Alcohol drinking (%)	28.0	24.7	0.327
Having exercise habit (%)	47.2	33.0	<0.001**
Comorbidity (%)	56.4	57.3	0.828
Frailty (%)	58.3	68.3	0.006**
Cognitive impairment (%)	69.0	78.6	0.004**


*Data are the mean ± SD unless otherwise indicated. Comorbidity was defined as the presence of two or more chronic diseases (hypertension, cardiovascular disease, cerebrovascular disease, diabetes, respiratory disease, digestive disease, chronic renal disease, and osteoarthritis). Abbreviations: BMI, body mass index; WC, waist circumference; SBP, systolic blood pressure; DBP, diastolic blood pressure; MMSE, mini-mental status examination. ^∗∗^*P* < 0.01.*

### The Relationship Between Frailty, Cognitive Impairment, and All-Cause Mortality

**FIGURE 1:**
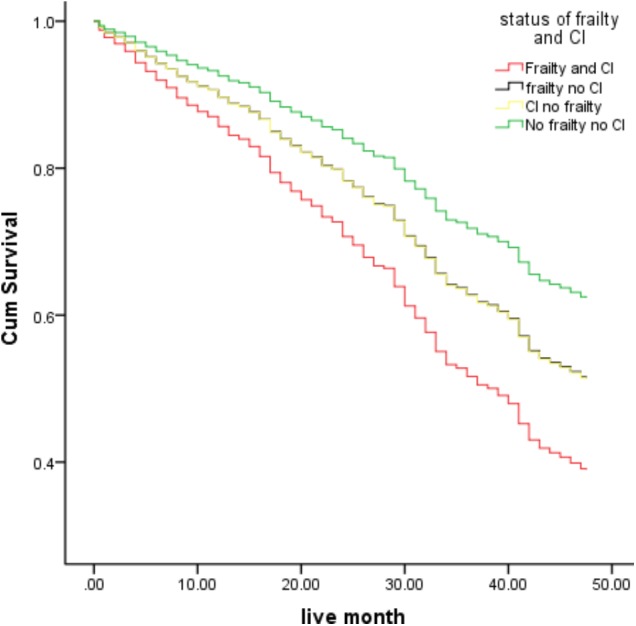
Survival curves of the study population, according to frailty and cognitive impairment status at baseline (the survival curves significantly differs in the Cox model when compared between the joined frailty and cognitive impairment vs. control group). CI: Cognitive impairment.

**Table 3** shows the results from unadjusted and adjusted Cox proportional hazard regression models for the frailty and cognitive impairment status, as a function of increased risk of death. Compared to the control group, subjects with combined frailty and cognitive impairment had a significantly higher risk of mortality [HR: 1.82, 95% CI (1.27, 2.61), *P* = 0.001] than those with the individual syndrome. Frailty only could not predict the risk of death in the study population [HR: 1.29, 95% CI (0.83, 2.00), *P* = 0.256] when compared with the control group. A similar result was yielded when participants with cognitive impairment alone were compared with the control group [HR: 1.31, 95% CI (0.88, 1.94), *P* = 0.184]. This model was stable after adjusting for age, gender, education levels, lifestyles, and other potential confounding factors. Subjects with joint frailty and cognitive impairment had a significantly higher risk of mortality, compared to the control group after adjustment of these potential confounding factors (HR: 2.13, 95% CI (1.39, 3.24), *P* < 0.001). Neither frailty alone nor cognitive impairment alone was able to predict the risk of mortality, as compared to the control group. **Figure 1** shows the survival curves of the study population according to their frailty and cognitive impairment status at baseline.

**Table 3:** Estimate of the effect of frailty and cognitive impairment mortality modeled with Cox regression model.

Models	Group	HR 95% CI	*P*-value
Unadjusted model	Frailty and cognitive impairment jointly	1.82 (1.27, 2.61)	0.001^∗∗^
	Frailty only	1.29 (0.83, 2.00)	0.256
	Cognitive impairment only	1.31 (0.88, 1.94)	0.184
	No frailty and no Cognitive impairment	1 (Reference)	N/A
Adjusted model 1^a^	Frailty and cognitive impairment jointly	2.06 (1.39, 3.04)	<0.001^∗∗^
	Frailty only	1.40 (0.90, 2.20)	0.136
	Cognitive impairment only	1.51 (0.99, 2.30)	0.058
	No frailty and no Cognitive impairment	1 (Reference)	N/A
Adjusted model 2^b^	Frailty and cognitive impairment jointly	2.00 (1.33, 3.00)	0.001^∗∗^
	Frailty only	1.41 (0.88, 2.25)	0.152
	Cognitive impairment only	1.41 (0.93, 2.16)	0.109
	No frailty and no Cognitive impairment	1 (Reference)	N/A
Adjusted model 3^c^	Frailty and cognitive impairment jointly	2.13 (1.39, 3.24)	<0.001^∗∗^
	Frailty only	1.49 (0.92, 2.42)	0.106
	Cognitive impairment only	1.43 (0.93, 2.20)	0.108
	No frailty and no Cognitive impairment	1 (Reference)	N/A


*^a^Adjusted for age, gender, and educational levels. ^*b*^Adjusted for factors in adjusted model 1 plus smoking, alcohol drinking, exercise habit, and comorbidity. ^*c*^Adjusted for factors in adjusted model 2 plus BMI, body mass index, height, weight, and systolic blood pressure (SBP). ^∗∗^*P* < 0.01.*

## Discussion

In this report, we studied the relationship between frailty, cognitive impairment, and mortality in community-dwelling oldest-old people (90–108 years) in Dujiangyan, Chengdu, and Sichuan province in China. Our present study is the first to investigate the combined role of frailty and cognitive impairment in predicting mortality among old people with advanced age. We have shown that the combined syndromes, and not frailty alone or cognitive impairment alone, is a significant risk factor for death among the oldest-old Chinese people. This study indicates that it is critical to assess a combination of frailty and cognitive function than as separate entities to predict the risk of mortality among old-age people, and also to define the existence of “cognitive frailty,” coined by the ICG in 2013.

The IANA and the IAGG organized the ICG in 2013 who first proposed the operational definition of cognitive frailty, described as the simultaneous presence of both physical frailty (Fried frailty phenotype) and cognitive impairment (clinical dementia rating [CDR] = 0.5) (Kelaiditi et al., 2013). The prevalence of cognitive frailty was estimated to be 1.0–1.8% among the community setting of old-age people without dementia or other neurodegenerative conditions, which suggested a limited clinical utility of cognitive frailty in the elderly (Sugimoto et al., 2018). Shimada et al. (2013) included 5104 older adults (mean age 71 years) in Japanese community studies and found that the prevalence of combined frailty and cognitive impairment was only 2.7%. However, our present study, which included Chinese non-agenarians and centenarians, revealed that the prevalence of combined frailty and cognitive impairment was 50.1% (95% CI = 46.4–53.8%), which indicated that cognitive frailty is more common in the very old population and supports the idea that the prevalence of the dual syndrome increases with age (Feng et al., 2017a). In other words, the simultaneous presence of both physical frailty and cognitive impairment is common among the oldest-old population.

The 34-item FI was employed to assess the presence of frailty in this study, while the frailty phenotype proposed by Fried et al. (2001) was most commonly used in previous studies (Zaslavsky et al., 2013). Additionally, the frailty phenotype was recommended to define cognitive frailty by the international consensus (Kelaiditi et al., 2013). To date, although multiple operational frailty assessment methods have been validated, frailty phenotype and FI are two most common measures of frailty (Cesari et al., 2014; Blodgett et al., 2015). Since we did not have data on grip strength and walking speed in our study, we could not use frailty phenotype to define frailty and to compare FI with frailty phenotype in predicting mortality. However, the operational definition of frailty in cognitive frailty also needed to be discussed. Although these two commonly used measures of frailty are different, both are associated with mortality and cognitive impairment (Cesari et al., 2014; Sugimoto et al., 2018). Furthermore, FI could be used to classify more people as frail, as it is based on a more comprehensive geriatric assessment, such as physical examinations, multi-functional measures, and diagnostic data, and hence, more capable of predicting mortality than frailty phenotype measurements (Theou et al., 2013; Cesari et al., 2014). Thus, it is rational to use FI to define frailty in the operation of cognitive frailty.

Although CDR = 0.5 was recommended to assess cognition in cognitive frailty by the international consensus (Kelaiditi et al., 2013), the majority of studies identified cognitive impairment according to global cognitive assessment scales, including MMSE and Montreal Cognitive Assessment (MoCA) (Sugimoto et al., 2018). Among these studies, MMSE was used to define cognitive impairment, focusing on the association of physical frailty with cognitive impairment, and the cut-off point varied from 18/30 to 26/30 (Matusik et al., 2012; Sugimoto et al., 2018). In contrast, our study, which focused specifically on very old cohort as most of the participants (97.4%) with low educational level (illiterate or primary school), yielded mean and median scores of MMSE equal to 14.82 ± 5.68 and 15, respectively. We set 18 as the cut-off point for cognitive impairment, according to previous studies (Matusik et al., 2012). In the Chinese population, this value has been shown to effect acceptable sensitivity (80–90%) and specificity (80–100%) for diagnosis of cognitive impairment (Katzman et al., 1988; Tombaugh and McIntyre, 1992; Zhu et al., 2006; Cui et al., 2011). Had we used 26 as the cut-off point for the MMSE score in our present study, the cognitive impairment would have been 97.9%. Therefore, we considered the MMSE = 18 as an acceptable cut-off point for the determination of diagnosis of cognitive impairment among the very old people. However, only using MMSE to assess cognitive function would be considered as one of the limitations in the present study.

It is a well-known fact that the risk of death increases exponentially with age during the human lifespan (Searle and Rockwood, 2015) and mortality is high among very old people. We obtained a 4-year death rate of 53.8% with our study participants, and it is the first study to analyze the combined effect of frailty and cognitive impairment in the oldest-old people. Our results are consistent with those of the previous studies conducted among old people in nursing homes by Matusik et al. (2012). Their study included 86 old people, living in two nursing homes, with ages ranging from 66 to 101 years (mean age: 83.8 ± 8.3 years). They predicted mortality (50.0%) of the combined frailty and cognitive function in a 1-year follow up, but did not find statistical significance between mortality with the separated syndromes among the disabled geriatric patients (Matusik et al., 2012). Our study extends the funding to community-dwelling of very old Chinese people, which might be related to the inconsistencies in reports from other groups (Jacobs et al., 2011; Forti et al., 2014). Forti et al. (2014) found that a clock drawing test other than frailty phenotype might predict the 7-year risk of all-cause mortality, but combining these two syndromes (frailty and cognitive impairment) did not improve the prognostic abilities among 766 dementia-free Italian community dwellers (mean age: 73.6 ± 5.9 years). On the other hand, Jacobs et al. (2011) found frailty phenotype other than cognitive impairment (assessed by MMSE) to be significantly predictive of 5-year mortality among 840 community-dwelling people with ages ranging from 85 to 90 years (Jacobs et al., 2011). In the present study we did not find blood pressure, smoking, or obesity to influence mortality, which did not support the evidence generated from other populations (Park et al., 2013; Pan et al., 2015). The differences of these observations might be explained by the differences in age groups, races, and follow-up periods. The potential mechanism of combining frailty and cognitive impairment in predicting mortality should be further investigated, using a large sample size, validated assessment methods, and reasonable follow-up period.

The results of our present study should be interpreted with caution for the following limitations. First, we included 705 participants (males: 230, females: 475) in this study, and the number of subjects is low in the control group (no frailty and no cognitive impairment group, *n* = 86). This might reduce the efficiency of statistical analysis and could limit the detection of the association of frailty or cognitive impairment and mortality. The small number of male participants also limited us from conducting subgroup analysis according to gender. However, the prevalence of frailty, cognitive impairment, and death ranked high in this specific cohort, which gave us the opportunity to examine these associations in a stable elderly population. Second, we only included Chinese Han oldest-old people, which is a good model to avoid major fatal diseases. However, this migh t have caused survival bias in our study, which could not be avoided. Moreover, we cannot extend the conclusion of our study to other races and it only could be extended to elderly people around the same age. Third, although we have adjusted age, gender, education level, and other potential confounding factors, all of the other potential confounders such as bilingualism, work life, neuropsychiatric or emotion issues, and family or social support may also play a role affecting both frailty and cognitive functioning with age. Fourth, most subjects (90%) in PLAD from a rural community were farmers who, now in their old age, would have usually had regular physical activities in their work age, limiting the extension of our study conclusion to the urban population. Fifth, we did not have data for the reason of death of our participants, so we cannot attribute only frailty and cognitive impairment to all-cause mortality in this study.

## Conclusion

Both frailty and cognitive impairment are very common among non-agenarians and centenarians. The combined syndrome, and not frailty or cognitive impairment alone, is a significant risk factor for death among the oldest-old Chinese people. This indicates that frailty and cognitive function should be assessed jointly other than separately in predicting mortality among the elderly population and defining cognitive frailty as essential for the prediction of mortality and assisted caregiving decisions for the elderly. Additionally, prospective studies with large sample size starting in middle age, and following up the participants in early old age (60–65 years) and then every 4–5 years, are warranted to inform about the mechanistic relationship between frailty and cognitive functions.

## Author Contributions

QH conducted the data analysis and drafted the initial manuscript. MY and Biao D helped with results interpretation and gave critical comments for the manuscript. Birong D and YW secured funding for data collection and verified the analysis outcomes. All authors read and approved the final manuscript.

## Conflict of Interest Statement

The authors declare that the sponsors of this research studies did not partake in the design, methods, data collection, analysis, or in the preparation of this manuscript.
